# Quantitative analysis of *HER2* mRNA expression by RNA *in situ* hybridization in canine mammary gland tumors: Comparison with immunohistochemistry analysis

**DOI:** 10.1371/journal.pone.0229031

**Published:** 2020-02-14

**Authors:** Byung-Joon Seung, Seung-Hee Cho, Soo-Hyeon Kim, Ha-Young Lim, Jung-Hyang Sur

**Affiliations:** Department of Veterinary Pathology, Small Animal Tumor Diagnostic Center, College of Veterinary Medicine, Konkuk University, Seoul, Republic of Korea; Colorado State University, UNITED STATES

## Abstract

Spontaneously occurring canine mammary gland tumors share many features with human breast cancer, including biological behavior and histologic features. Compared to transgenic murine model, canine models have advantages including naturally occurring models of human diseases and cancer. In humans, breast cancer is divided into molecular subtypes based on ER, PR, and *HER2* expression. In contrast with humans, few studies have evaluated these subtypes in canine mammary gland tumors, including expression of *HER2*. *HER2* expression in canine mammary tissues has been further complicated by controversy regarding the antibody’s specificity. This study aimed to investigate *c-erbB2* mRNA expression in retrospective formalin-fixed paraffin embedded samples, using RNA *in situ* hybridization with a novel quantitative assay and to compare this method with immunohistochemistry. Using 48 canine mammary tumor samples and 14 non-neoplastic canine mammary tissues, RNA *in situ* hybridization was performed with RNAscope^®^ using a canine-specific target gene probe (*ERBB2*), and quantitative measurement was performed using the housekeeping gene (*POLR2A*) to calculate the target gene/housekeeping gene ratio. The ratio of *ERBB2/POLR2A* was quantified using open-source image analysis programs and compared with the immunohistochemistry results. A significant correlation was observed between the *HER2* immunohistochemistry score and *ERBB2/POLR2A* RNA *in situ* hybridization (*P* < 0.001). When the *HER2* immunohistochemistry score was 3+, significantly higher expression of *HER2* mRNA was observed by RNA *in situ* hybridization. Interestingly, *HER2* mRNA was also observed in non-neoplastic mammary tissues by RNA *in situ* hybridization. This assay potentially facilitates the reliable quantification of mRNA expression levels in retrospective formalin-fixed paraffin-embedded samples. Further studies are required to elucidate the role of *HER2* in canine mammary gland tumors and to implement clinical trials in dogs.

## Introduction

Spontaneously occurring canine mammary gland tumors (CMTs) are the most common tumor type in intact female dogs [1, 2]. CMTs in dogs share many epidemiological, biological, and clinical features with human breast cancer including their biological behavior and histologic features [3]. The few actively used prognostic factors for CMTs include histopathological classification and histologic grading, which have now been modified to model the criteria for human breast cancer [4–6]. Unlike that in humans, in dogs, surgery is the main treatment option for CMTs, and other systemic treatment options are limited to the research stage because they have not been sufficiently studied [7, 8]. Therefore, further studies are required to provide a basis for treatments including chemotherapy for CMTs.

In humans, breast cancer exhibits well-established intrinsic subtypes (luminal A, luminal B, *HER2*-enriched, and basal-like), facilitating accurate diagnosis and effective treatment [9]. Among these, the *HER2*-enriched subtype, accounting for approximately 15–25% of breast cancer cases, benefits from *HER2*-targeted chemotherapy using trastuzumab in humans [10–12]. In human breast cancer, *HER2* status was commonly determined using Immunohistochemistry (IHC) or fluorescence *in situ* hybridization [13]. Few studies, however, have evaluated the molecular subtypes of CMTs by immunohistochemistry, including *HER2* expression, and have revealed inconsistent results [14, 15]. Ahern *et al*. reported that *HER2* mRNA levels were lower in benign CMTs than in malignant CMTs through hybridization of total polysomal RNA with the human *c-erbB-2* probe [16]. However, Peña *et al*. reported discordant results in subsequent IHC studies [17]. Additionally, studies of *HER2* expression in CMTs using IHC with an FDA-approved anti-*HER2* polyclonal antibody (A0485, Dako, Glostrup, Denmark) revealed differences in the expression patterns and non-specific cytoplasmic staining patterns in accordance with the criteria for human breast cancer [18, 19].

RNAscope is a recently developed method for RNA *in situ* hybridization (RNA-ISH), using a novel probe design and unique amplification system to amplify target-specific signals without background interference [20]. This RNA-ISH technique can be used to rapidly detect RNA with high sensitivity in formalin-fixed paraffin-embedded (FFPE) tissues [20].

In this study, we investigated *HER2* mRNA levels by assessing *c-erbB2* expression in CMTs using RNA-ISH with a new quantitative assay method in retrospective FFPE CMTs samples. We assessed *HER2* protein levels in CMTs by immunohistochemistry using the FDA-approved anti-*HER2* antibody and compared the results with those obtained using RNA-ISH.

## Materials and methods

### Ethical statement

The protocol for tissue sampling was approved by the Institutional Animal Care and Use Committee of Konkuk University (KU16106, KU17162, and KU18168). Tissue samples were acquired as routine diagnostic procedures from privately owned pet dogs via private veterinary hospitals with informed consent from the owner.

### Case selection and histopathological analysis

Forty-eight CMT samples and 14 non-neoplastic canine mammary tissue samples that were suspected tumors but diagnosed as mammary gland hyperplasia were selected from the archived FFPE database from 2017 to 2019 at the Department of Veterinary Pathology, Konkuk University. Simple random sampling was performed for CMT samples yielding *HER2* IHC data (available from our previous data descriptor [21] and validation studies) with complete clinical data. During RNA-ISH, tissue samples not suitable for analysis were excluded (describe in detail below). To prevent unequal distribution of the *HER2* IHC score in malignant CMTs, additional selections were performed until each *HER2* IHC score (1+, 2+, and 3+) was obtained from at least 10 samples. Ultimately, 38 FFPE CMT specimens were included in our previous data descriptor article [21].

Forty-three dogs were intact females and 19 dogs were spayed females. The breeds included Maltese (n = 20), Shih-Tzu (n = 10), Mixed (n = 9), Poodle (n = 8), Schnauzer (n = 5), Yorkshire Terrier (n = 3), Cocker Spaniel (n = 2), Pomeranian (n = 2), English sheepdog (n = 1), Miniature Pinscher (n = 1) and Pekingese (n = 1). The age range of the dogs was 2–16 years [mean ± standard deviation (SD): 11.15 ± 3.02]. The detailed regarding sample characteristics are listed in S1 Table.

For histological examination, 4-μm-thick sections from FFPE tissues were stained with hematoxylin and eosin and diagnosed by two researchers (B.J.S. and J.H.S.). The histological subtype of each sample was categorized based on the World Health Organization classification for CMTs [4]. The histological grade was assessed based on the criteria described by Peña *et al*. [6]. Lymphatic invasion, defined as infiltration of tumor cells in lymphatic vessels, was also evaluated.

### Immunohistochemistry

To evaluate the expression of *HER2* protein, 4-μm-thick sections from FFPE tissues were used. Because of intra-tumor heterogeneity, serial sections were used for IHC and ISH experiments. IHC was performed as previously described with the polyclonal anti-human c-erbB-2 oncoprotein antibody (Dako) [22]. Control slides known to be positive for *HER2* were used as positive controls. Isotype-matched immunoglobulins were used as negative controls. *HER2* staining was scored on the basis of the guidelines of the American Society of Clinical Oncology/College of American Pathologists (0: No staining is observed or incomplete membrane staining that is faintly perceptible within ≤10% of epithelial tumor cells; 1+: Incomplete membrane staining that is faintly perceptible and within >10% of epithelial tumor cells; 2+: Weak to moderate complete membrane staining within >10% of tumor cells or complete intense membrane staining within ≤10% of tumor cells; and 3+: Intense complete membrane staining within >10% of tumor cells) [23]. According to human criteria, the observation of cytoplasmic staining (described by Burrai et al [19]) is non-specific, and the evaluation by B.J.S. was focused on a complete membranous staining pattern between score 1+ and 2+ and staining intensity between score 2+ and 3+. For ambiguous samples, a consensus was reached by the two researchers (B.J.S. and J.H.S.).

### RNA *in situ* hybridization

RNA-ISH was performed for FFPE tissues using the RNAscope duplex assay (Advanced Cell Diagnostics, Hayward, CA, USA). Because the RNA quality of FFPE tissues retrieved from storage archives showed variation following the storage period and fixation process [24], we performed quantitative analysis through dual detection of target genes and housekeeping genes in one section and determined the target gene/housekeeping gene ratio. To detect the target mRNA (*HER2*) in individual epithelial cells, we used an *ERBB2* target probe (Cat. No.432411, Advanced Cell Diagnostics). A *POLR2A* probe (Cat. No.479111-C2, Advanced Cell Diagnostics) was used as a housekeeping gene following the manufacturer’s recommendations for canine tissue. GenBank accession numbers and probe regions are as follows: *ERBB2* (GenBank, NM_001003217.1; probe region, 1585–2823) and *POLR2A* (GenBank XM_852751.3; probe region, 1846–2924). The procedure was manually carried out in accordance with the manufacturer’s instructions. Briefly, 4-μm-thick sections (serial sections with IHC slides) were baked for 1 h at 60°C in an oven, deparaffinized in xylene twice for 5 min each, and dehydrated in 100% ethanol twice for 2 min each. After the sections were air-dried, they were treated with RNAscope hydrogen peroxide solution (Cat. No. 322330, Advanced Cell Diagnostics) for 10 min at room temperature and washed with distilled water. The sections were incubated in target retrieval reagent (Cat. No.322000, Advanced Cell Diagnostics) maintained at a boiling temperature (93–98°C) using a hot plate for 15 min, and then washed with distilled water. A hydrophobic barrier was drawn around the samples using an Immedge hydrophobic barrier pen (Cat. No. H-4000, Vector Laboratories, Burlingame, CA, USA). Each section was treated with Protease plus (Cat. No.322330, Advanced Cell Diagnostics) reagents for 30 min at 40°C in a HybEZ hybridization oven (Advanced Cell Diagnostics). The sections were then incubated for 2 h at 40°C in a HybEZ hybridization oven using probes mixed with an *ERBB2* probe (C1-Blue) and *POLR2A* probe (C2-red) at a 50:1 ratio. The slides were repeatedly washed twice with wash buffer reagent (Cat. No.310091, Advanced Cell Diagnostics) after each amplification step using RNAscope 2.5 HD Duplex Detection Reagent (Cat. No.322500, Advanced Cell Diagnostics). Chromogenic detection was carried out using fast red (C2), followed by DAB chromogenic (C1) detection for 10 min at room temperature. Counterstaining was performed using 50% Gill’s hematoxylin. The bacterial gene DapB probe was used as a negative control at the same mixing ratio (50:1) for the C1 and C2 probe.

### Evaluation of RNA-ISH results

**Fig 1:**
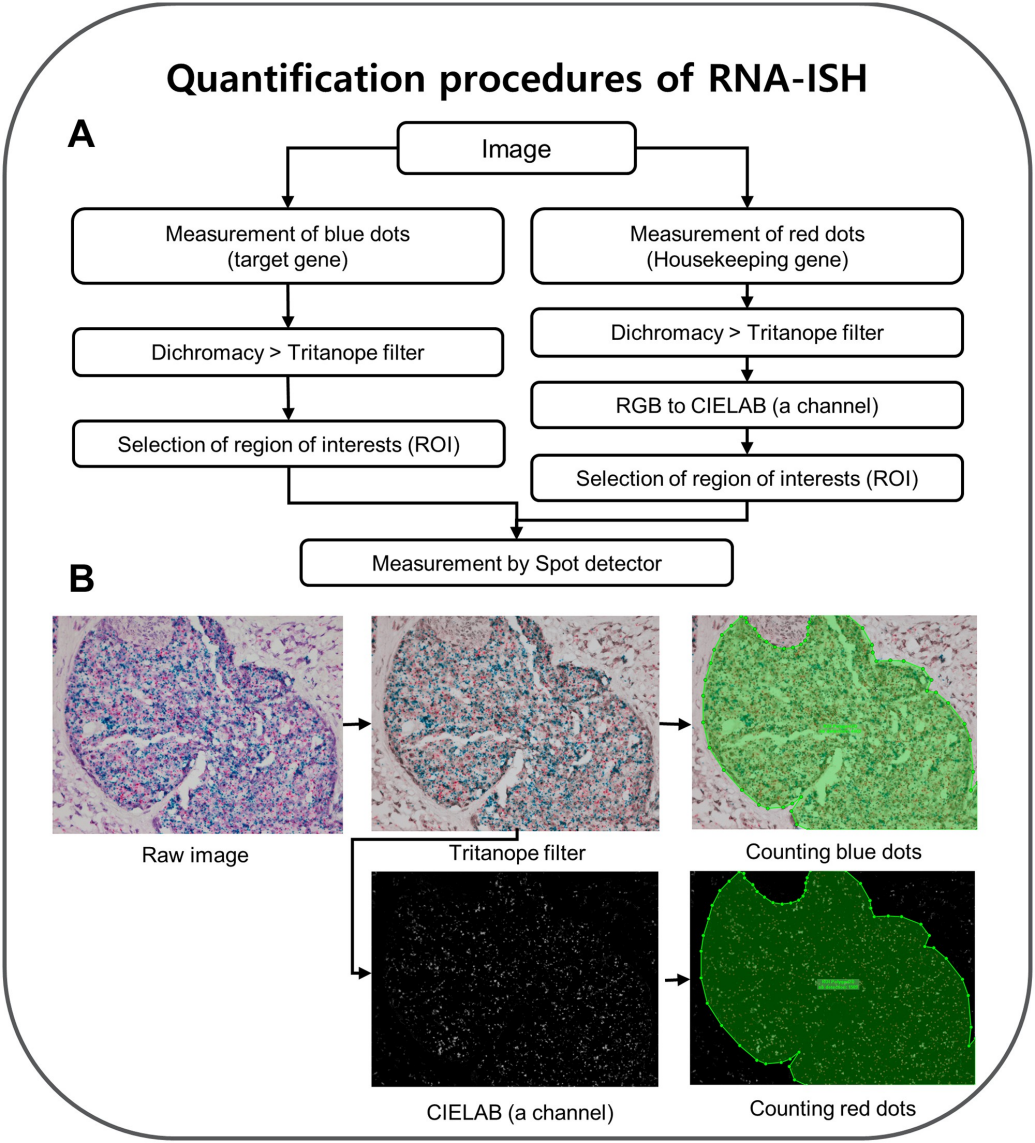
Workflow of quantification procedures of RNA-ISH. (A) Flow chart for quantitative analysis of RNA-ISH images. (B) Representative RNA-ISH raw images are converted using each filter, and quantitative analysis is automatically performed using the spot detection software.

**Fig 2:**
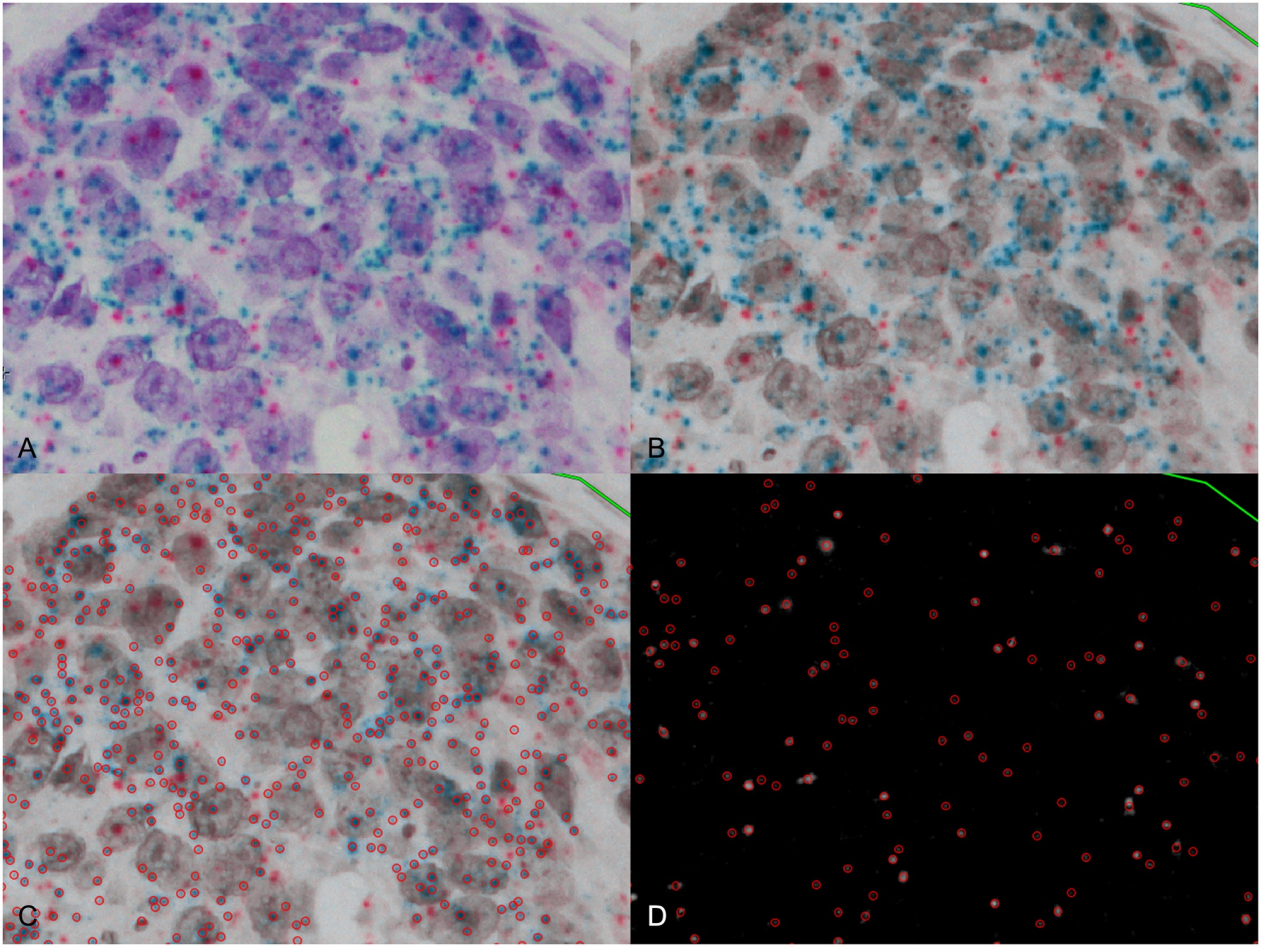
Example of analyzing RNA-ISH images. (A) Original image of RNA-ISH. Blue dots (*HER2*) and red dots (*POLR2A*) were observed. (B) Blue dots and red dots were observed in Tritanope filtered image. (C) Blue dots are recognized and enumerated by spot detector of open-source program. (D) Red dots are recognized and enumerated by spot detector of open-source program.

RNA-ISH images were acquired (by B.J.S.) from five representative regions corresponding to the IHC scores and the histological diagnosis at 400× magnification for each sample. Digital images were acquired using an Olympus BX51 microscope (Tokyo, Japan) and Image transfer software (Olympus). In the RNA-ISH results, blue dots (*HER2* mRNA) and red dots (*POLR2A* mRNA) were measured to determine the average ratios (*ERBB2*/*POLR2A* ISH ratio) for five representative images per sample. We used two open-source image analysis programs (Fiji [25] and ICY [26]) to analyze the images obtained after RNA-ISH experiments. First, the images were converted using the “Dichromacy > Tritanope” filters in Fiji to select blue signals against hematoxylin counterstaining. After selecting regions of interest (ROI) containing only epithelial regions in the filtered image (400× magnification; by B.J.S.), using ICY program, blue dots (*ERBB2* mRNA) were measured using dark spot detection mode in the spot detector of ICY. To measure the red dots, Tritanope-filtered images were converted to a CIELAB (RGB to CIELAB) and ‘a’ channel images were acquired as jpg files. Red dots (*POLR2A* mRNA) were quantified using bright spot detection mode in the spot detector of ICY. In some tissue sections, the red channel (housekeeping gene) was not successful owing to RNA degradation or observed with high background of fast red may be due to endogenous alkaline phosphatase. These samples were excluded from the analysis. The quantification procedures are illustrated in Figs 1 and 2. And quantification procedure also deposited at protocols.io (dx.doi.org/10.17504/protocols.io.badcia2w).

### Statistical analysis

The *ERBB2*/*POLR2A* ISH ratio and *HER2* IHC score were compared by the Kruskal–Wallis test, followed by post-hoc analysis with Bonferroni correction. Comparisons between ISH ratio and three groups were also performed using Kruskal-Wallis test and comparisons between ISH ratio and two groups were performed using Mann–Whitney U test. Categorical variables were analyzed using Fisher’s exact test. Statistical analyses were performed using SPSS version 24.0 software for Windows (SPSS, Inc., Chicago, IL, USA). Values were considered as significant when *P* < 0.05.

## Results

### Histology

Histologically, samples were classified as malignant (n = 42), benign (n = 6) and hyperplasia (n = 14). The histological subtypes of CMTs were classified as simple adenoma (n = 4), complex adenoma (n = 2), simple carcinoma (n = 26), and complex carcinoma (n = 16). The histological grade of malignant CMTs included grade 1 (n = 23), grade 2 (n = 9), and grade 3 (n = 10). Six cases of malignant CMTs exhibited evidence of lymphatic invasion.

### *HER2* protein expression

Among the 62 CMT and non-neoplastic tissue samples, the *HER2* scores were classified as 1+ (n = 20), 2+ (n = 24), and 3+ (n = 18). In malignant CMT samples, 15 cases of carcinoma (8 simple and 7 complex) were scored as 1+, 15 cases of carcinoma (10 simple and 5 complex) were scored as 2+, and 12 cases of carcinoma (8 simple and 4 complex) were scored as 3+, with intense and complete immunoreactivity observed in more than 10% of epithelial tumor cells. In benign CMT samples, 2 cases of adenoma (2 complex) were scored as 1+, 3 cases of adenoma (3 simple) were scored as 2+, and 1 case of adenoma (1 simple) was scored as 3+. In mammary gland hyperplasia samples, 3 cases of samples were scored as 1+, 6 cases of samples were scored as 2+, and 5 cases of samples were scored as 3+. The results of the *HER2* IHC score according to histological subtype were summarized in Table 1. In line with other studies [18, 19, 27], we also observed the cytoplasmic staining patterns of *HER2* in CMTs, and only membranous staining patterns were evaluated in this study. *HER2* expression was also observed in adjacent normal or hyperplastic mammary glands in CMTs and non-neoplastic tissues.

**Table 1:** The *HER2* IHC score according to histological subtype of samples.

	Histological subtype				
	Non-neoplastic tissue	Benign CMT		Malignant CMT	
	Mammary gland hyperplasia	Simple adenoma	Complex adenoma	Simple carcinoma	Complex carcinoma
*HER2* IHC Score	n/total (%)	n/total (%)	n/total (%)	n/total (%)	n/total (%)
**0**	0/0 (0%)	0/0 (0%)	0/0 (0%)	0/0 (0%)	0/0 (0%)
**1+**	3/20 (15.0%)	0/20 (0%)	2/20 (10.0%)	8/20 (40.0%)	7/20 (35.0%)
**2+**	6/24 (25.0%)	3/24 (12.5%)	0/24 (0%)	10/24 (41.7%)	5/24 (20.8%)
**3+**	5/18 (27.8%)	1/18 (5.6%)	0/18 (0%)	8/18 (44.4%)	4/18 (22.2%)

IHC, Immunohistochemistry; CMT, Canine mammary gland tumor

### *HER2* mRNA expression

*HER2* mRNA expression in canine mammary gland tissue was investigated by the RNA-ISH method. Because retrospective FFPE tissue samples from archives contain different amounts of RNA, the ratio between *ERBB2* and the reference gene (*POLR2A*) was determined. The range of the *ERBB2*/*POLR2A* ISH ratio was 1.521–4.952 [mean ± standard deviation (SD): 3.079 ± 0.743]. The association between the *HER2* (RNA-ISH results and IHC results) and parameters including the histological diagnosis, histological subtype, malignancy, histological grade, and presence of lymphatic invasion are summarized in Tables 2 and 3. *HER2* mRNA dots were primarily observed in epithelial regions of the tumor. Although *POLR2A* reference mRNA dots were observed in both the epithelial and stromal regions, only the epithelial regions were counted upon selecting the ROI. In samples with upregulated *HER2* mRNA, the mRNA dots were observed as clusters and dense clusters of *HER2* mRNA signals were counted less than actual mRNA expression in some 3+ score cases. (S1 Fig).

**Table 2:** Association between the *ERBB2/POLR2A* ISH ratio and parameter including malignancy, histological subtype, histological grade, presence of lymphatic invasion, and *HER2* IHC score.

	*ERBB2/POLR2A* ISH ratio (Mean ± SD)	*P*-value
**Histological diagnosis** ^a^		
Non-neoplastic lesion (n = 14)	3.33 ± 0.80	0.301
Benign (n = 6)	2.78 ± 0.21	
Malignant (n = 42)	3.04 ± 0.76	
**Histological subtype** ^b^		
Simple type (n = 30)	3.02 ± 0.77	0.983
Complex type (n = 18)	2.98 ± 0.64	
**Histological grade** ^a^		
Grade 1 (n = 23)	2.90 ± 0.59	0.174
Grade 2 (n = 9)	2.89 ± 0.89	
Grade 3 (n = 10)	3.47 ± 0.90	
**Lymphatic invasion** ^b^		
Absent (n = 36)	2.99 ± 0.72	0.428
Present (n = 6)	3.30 ± 0.97	
***HER2* IHC score in malignant CMTs** ^a^		
1+ (n = 15)	2.43 ± 0.56	**<0. 001**
2+ (n = 15)	3.01 ± 0.40	
3+ (n = 12)	3.82 ± 0.62	
***HER2* IHC score in canine mammary tissues** ^a^		
1+ (n = 20)	2.51 ± 0.52	**<0. 001**
2+ (n = 24)	3.00 ± 0.50	
3+ (n = 18)	3.81 ± 0.62	
***HER2* membranous staining pattern in malignant CMTs** ^b^		
Incomplete (1+) (n = 15)	2.43 ± 0.56	**< 0.001**
Complete (2+ and 3+) (n = 27)	3.37 ± 0.65	
***HER2* membranous staining pattern in canine mammary tissues** ^b^		
Incomplete (1+) (n = 20)	2.51 ± 0.52	**< 0.001**
Complete (2+ and 3+) (n = 42)	3.35 ± 0.68	

IHC, Immunohistochemistry; CMTs, Canine mammary gland tumors; SD, Standard deviation

^a^ Kruskal-Wallis test

^b^ Mann-Whitney test

**Table 3:** Association between *HER2* IHC score and parameter including histological diagnosis, histological grade, presence of lymphatic invasion.

	*HER2* 1+ (IHC) (Incomplete membranous staining)	*HER2* 2+ and 3+ (IHC) (Complete membranous staining)	*P*-value
	n/total (%)	n/total (%)	
**Histological Diagnosis** ^a^			
Non-neoplastic (n = 14)	3/14 (21.4%)	11/14 (78.6%)	0.697
Benign CMTs (n = 6)	2/6 (33.3%)	4/6 (66.7%)	
Malignant CMTs (n = 42)	15/42 (35.7%)	27/42 (64.3%)	
**Histological grade** ^a^			
Grade 1 (n = 23)	8/23 (34.8%)	15/23 (65.2%)	0.317
Grade 2 (n = 9)	5/9 (55.6%)	4/9 (44.4%)	
Grade 3 (n = 10)	2/10 (20.0%)	8/10 (80.0%)	
**Lymphatic invasion** ^a^			
Absent (n = 36)	14/36 (38.9%)	22/36 (61.1%)	0.395
Present (n = 6)	1/6 (16.7%)	5/6 (83.3%)	

IHC, Immunohistochemistry

^a^ Fisher’s exact test

### Comparison of *HER2* expression by RNA-ISH and immunohistochemistry

**Fig 3:**
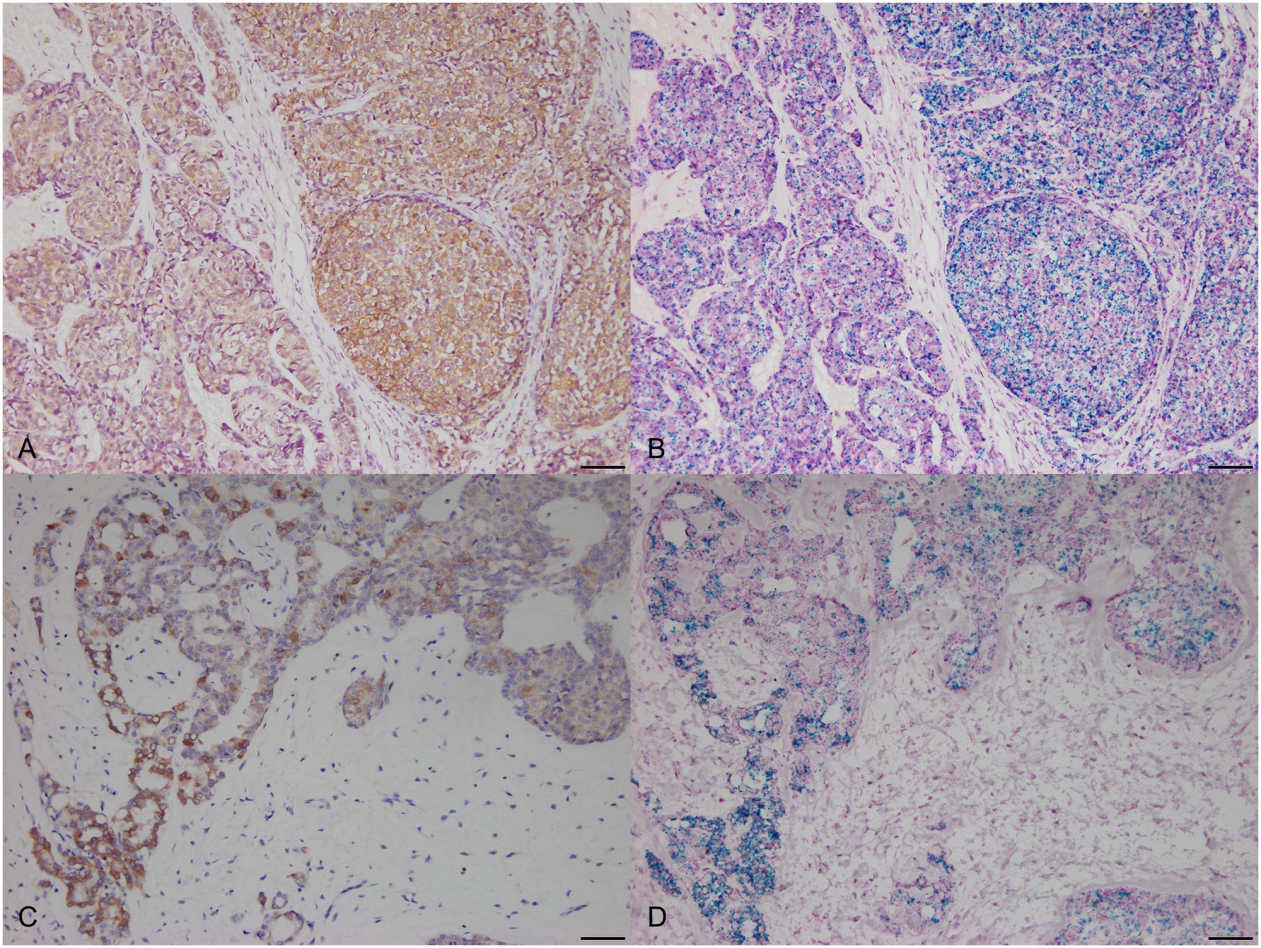
*HER2* expression in adjacent serial section of canine mammary gland tissues. (A) Strong membranous staining pattern was observed in the right side of images by IHC 200×, bar = 50 μm. (B) Expression of *HER2* mRNA dots was observed in corresponding regions of adjacent serial section to Fig 3A by RNA-ISH, 200×, bar = 50 μm. (C) Strong membranous staining pattern staining pattern was observed in the bottom-left side of images by IHC 200×, bar = 50 μm. (D) Expression of *HER2* mRNA dots was observed in corresponding regions adjacent serial sections to Fig 3C by RNA-ISH, 200×, bar = 50 μm.

**Fig 4:**
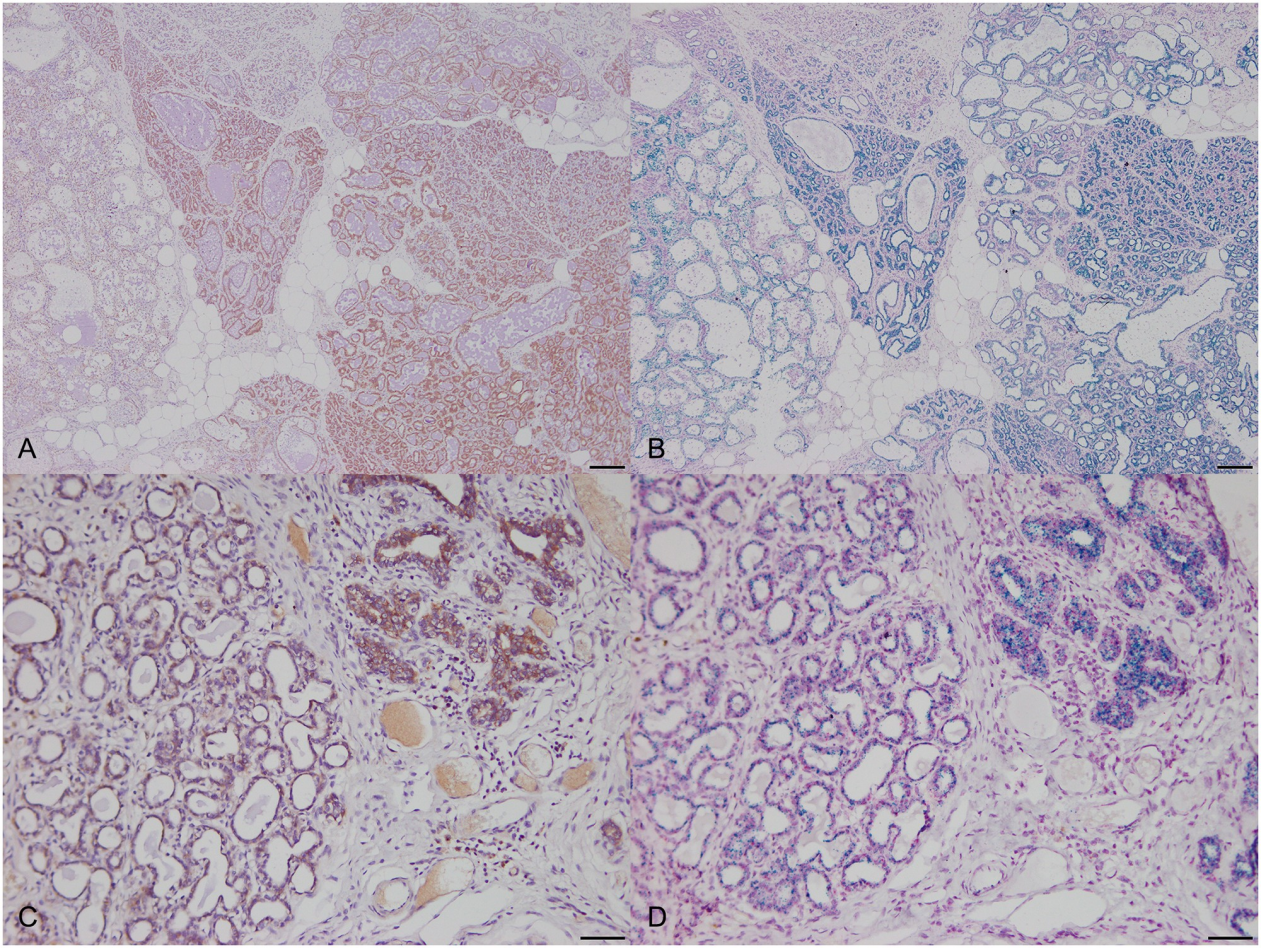
*HER2* expression in adjacent serial section of non-neoplastic regions. (A) Strong membrane staining pattern (3+) in right sides of mammary gland hyperplasia samples by IHC, 40×, bar = 200 μm. (B) Expression of *HER2* mRNA dots was observed in the section of serial to Fig 4A by RNA-ISH, 40×, bar = 200 μm. (C) Moderate *HER2* staining was observed in the adjacent normal mammary gland in CMT by IHC, 200×, bar = 50 μm. (D) Expression of *HER2* mRNA dots was observed in the section of serial to Fig 4C by RNA-ISH, 200×, bar = 50 μm.

**Fig 5:**
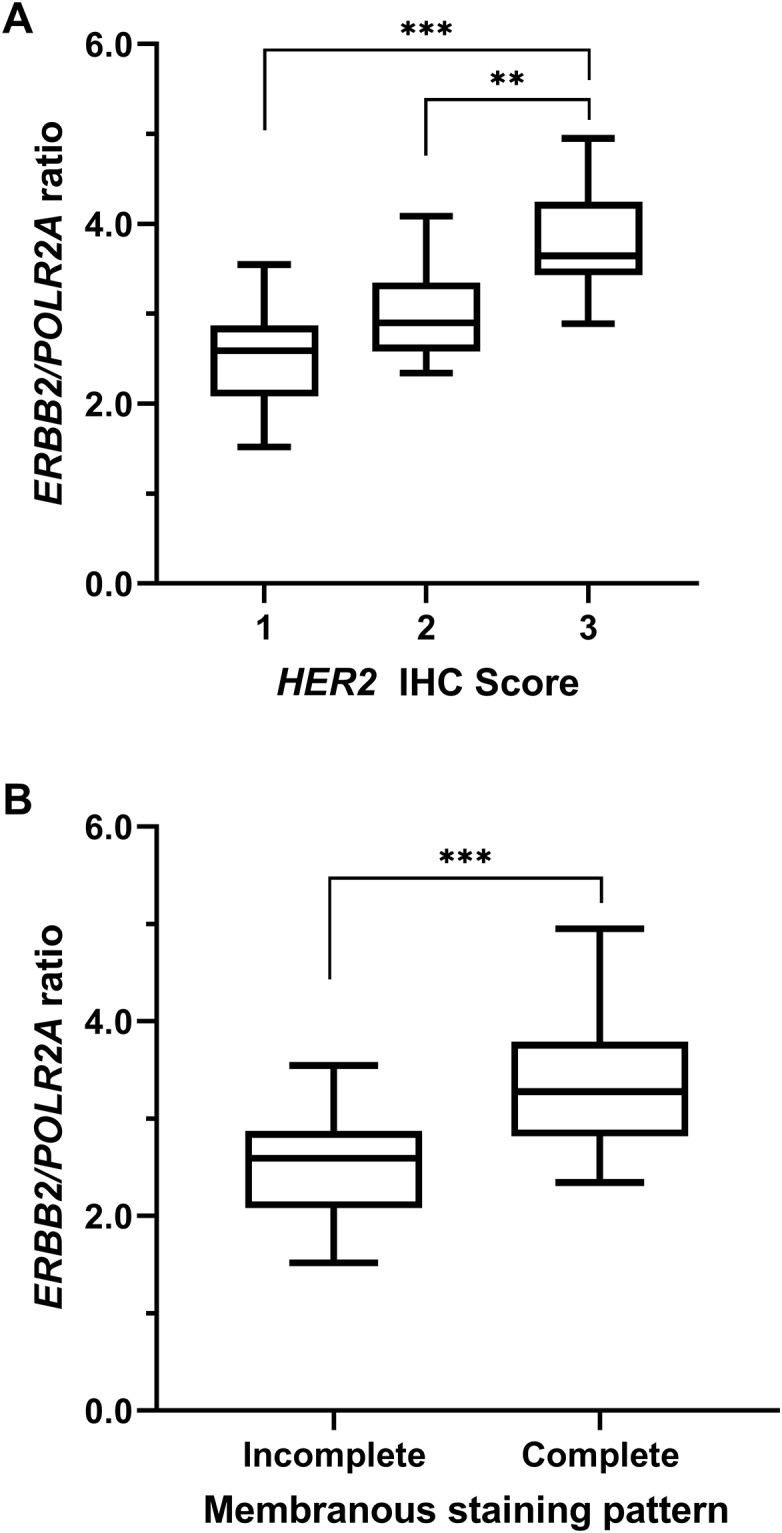
Comparison of the *ERBB2/POLR2A* ISH ratio according to *HER2* IHC score in canine mammary tissues. A significant correlation between the *HER2* IHC score and *ERBB2/POLR2A* ISH ratio was observed (**, *P* < 0.01; ***, *P* < 0.001).

To compare *HER2* IHC scores with the RNA-ISH results, RNA-ISH images were acquired in representative regions corresponding to those used to determine IHC scores. Expression of *HER2* mRNA dots was observed in the region showing a strong membrane staining pattern in IHC (Fig 3A–3D). Furthermore, the expression of *HER2* mRNA dots was observed in non-neoplastic lesions (Fig 4A and 4B). In some cases, the expression of *HER2* mRNA dots was observed in the area around the tumor where staining pattern was observed in the adjacent normal mammary gland by IHC (Fig 4C and 4D). A significant correlation between the *HER2* IHC score and *ERBB2/POLR2A* ISH ratio was observed (*P* < 0.000) in canine mammary tissues (Fig 5A and 5B). In addition, significant differences were observed between the 1+ score vs. 3+ score (Bonferroni-corrected *P* value < 0.001) and 2+ score vs. 3+ score (Bonferroni-corrected *P* value = 0.002) (Fig 5A). When the *HER2* IHC score was 3+, significantly higher expression of *HER2* mRNA was observed by RNA-ISH. Significant differences were observed based on complete membranous staining pattern between 1+ score (incomplete membranous staining) and 2+, 3+ score (complete membranous staining) (*P* < 0.001) (Fig 5B). No significant differences were observed in the *HER2* IHC score and RNA-ISH results for clinicopathological parameters.

## Discussion

In humans, breast cancers with *HER2* overexpression display aggressive behavior and are associated with reduced patient survival [28, 29]. Anti-*HER2* therapies including trastuzumab, a monoclonal antibody targeting the extracellular domain of *HER2*, has improved treatment outcomes [12, 30]. *HER2* protein has gained increasing attention in veterinary oncology and was recently investigated in dogs. However, studies on *HER2* expression in CMTs using human anti-*HER2* antibody, have shown variable results [14, 15, 31]. The specificity of human anti-*HER2* antibody (Dako A0485) for detecting *HER2* in canine tissues remains controversial. One study showed no evidence of its specificity in canine tissues by western blotting and subsequent mass spectrometric analysis [19], while another recent study showed the cross-reactivity of the human anti-*HER2* antibody in canine tissue by western blotting [27].

Furthermore, the present results displayed cytoplasmic immunoreactivity in tumor cells and non-neoplastic cells, similar to the results of some previous studies [18, 19, 27]. As this cytoplasmic immunoreactivity is considered non-specific in accordance with human criteria, we limited our analysis to membranous immunoreactivity. Previous studies [19, 32] have shown that mRNA and IHC levels are not completely correlated in CMTs, probably owing to post-translational events [33].

In our study, however, *HER2* mRNA levels were high in IHC samples with strong complete membranous staining pattern (3+), and a significant correlation was observed between the IHC score and RNA-ISH results. Furthermore, similar expression pattern was observed in high power field images (Figs 3 and 4) in which left and right sides were expressed differently due to intra-tumor heterogeneity [34]. This discrepancy in our results relative to previous results may have occurred by using consecutive sections and restricting RNA-ISH analysis of gene expression to epithelial regions and matching the IHC scores to prevent intra-tumor heterogeneity. Furthermore, this *in situ* detection technology (RNA-ISH) is superior to RT-PCR, which depends on an admixture of cells with a potentially low content of malignant epithelial cells. Our results are consistent with those of a previous study that analyzed *HER2* mRNA expression in human breast cancer by RT-PCR and RNA-ISH in parallel [35]. Significant differences in the RNA-ISH results were also observed depending on complete membranous staining pattern. Although the specificity of the anti-*HER2* antibody is controversial, the present results reduced the likelihood of nonspecific findings with antibodies during IHC by morphologically confirming mRNA expression directly in tissue and comparing it with IHC results. And thus, IHC method may be used as a complement to assess the *HER2* status determined from membrane staining pattern (RNA-ISH method is more specific).

To confirm the association between *HER2* expression and its prognosis in CMTs, we assessed the available clinical parameters. However, no significant correlation was observed between the *HER2* status (mRNA and protein levels) and clinical parameters (malignancy, histological subtype, histological grade, lymphatic invasion). This is consistent with the results of previous studies showing that *HER2* is not associated with poor prognostic factors in CMTs [18, 36, 37]. Additionally, present study revealed that HER2 is also expressed in non-neoplastic mammary gland tissues. This is a distinct characteristic from that of human breast cancer, suggesting prospects for future study from the viewpoint of comparative medical research. Recently, lower prevalence of *ERBB2* copy number variant was observed in HER2 protein overexpressing canine urothelial carcinoma [38]. In present study, we did not confirm the *HER2* amplification status in canine mammary tissues, which is correlated with *HER2* overexpression in human breast cancer, and only observed mRNA and protein levels; thus, further studies are needed to determine expression of *HER2* mRNA is due to gene amplification or other mechanism.

This study shows the use of quantitative RNA-ISH. RNA-ISH technology with RNAscope has already been used to for human breast cancer to determine *HER2* mRNA levels in equivocal cases (IHC 2+ or a Fluorescence ISH ratio of 1.8 to 2.2) and has displayed better performance than that by qPCR [35]. In retrospective studies, RNA preservation may vary between FFPE samples from archives; hence, we used a dual detection RNA-ISH method to compare with housekeeping genes, unlike that in previous studies. This assay method has been primarily used in fluorescence ISH to confirm *HER2* gene amplification by *HER2* to chromosome 17 ratio in breast cancer [39]. In addition, manual enumeration of dots is difficult; hence, to enumerate the dots to determine the target gene/housekeeping gene ratio, we developed an automated measurement protocol using open-source programs. The method displayed adequate performance without human subjectivity in recognizing dots. Although dense clusters of dots tended to be enumerated at lower levels than the actual expression levels, our experimental groups yielded significant results. The present method of *in situ* analysis of RNA in tissues is potentially applicable in studies using retrospective FFPE samples. Furthermore, the RNA-ISH method may be used to supplement existing IHC methods if the antibody is not adequately sensitive or if no antibodies are available (newly discovered gene signatures studies).

Compared to the use of transgenic murine models, the use of naturally occurring canine models of human diseases for translational research has benefits for dogs and for study cancer in humans [40]. Using canine models to evaluate human diseases is advantageous because they exhibit naturally occurring cancer, clinical similarities, high incidences, and shorter lifespans than humans [41, 42]. Recently, *HER2*-targeted cancer immunotherapy using recombinant *Listeria monocytogenes*, which expresses a chimeric human *HER2*/neu construct, was shown to prolong the overall survival and reduce metastasis rates in canine models of pediatric osteosarcomas [43]. Additionally, small-molecule tyrosine kinase inhibitor (lapatinib) of *HER2*, which may cross-react between species, exerted anti-tumor effects in canine transitional cell carcinoma cell lines [44]. Such comparative studies using canine models may not only facilitate individual therapeutic methods for dogs but are also applicable in understanding the pathogenesis of human cancers.

## Conclusions

In this study, we assessed *HER2* mRNA levels in CMTs using RNA-ISH and observed a correlation with the *HER2* immunohistochemistry score. We developed an automated, quantitative dual staining RNA-ISH method to evaluate *HER2* expression relative to that of a housekeeping gene. This assay potentially allows for reliable quantification of mRNA expression levels in retrospective FFPE samples. Because no correlation was identified in this study between *HER2* expression and clinical parameters, further studies are needed to clarify if there is a role of *HER2* in the pathogenesis of CMTs.

## Supporting information

S1 Table Clinical information of the samples. The avaialbe clinical information of 62 samples are listed.
(XLSX)

S1 Fig Example image of dense clusters of *HER2* mRNA dots tended to be enumerated at lower levels than the actual expression levels. (TIF)
