# Supplementary figures and images for: Quantitative analysis of HER2 mRNA expression by RNA in situ hybridization in canine mammary gland tumors: Comparison with immunohistochemistry analysis
Source: PLoS One. 2020 Feb 14;15(2):e0229031. doi: 10.1371/journal.pone.0229031 (PMC7021316; doi:10.1371/journal.pone.0229031)

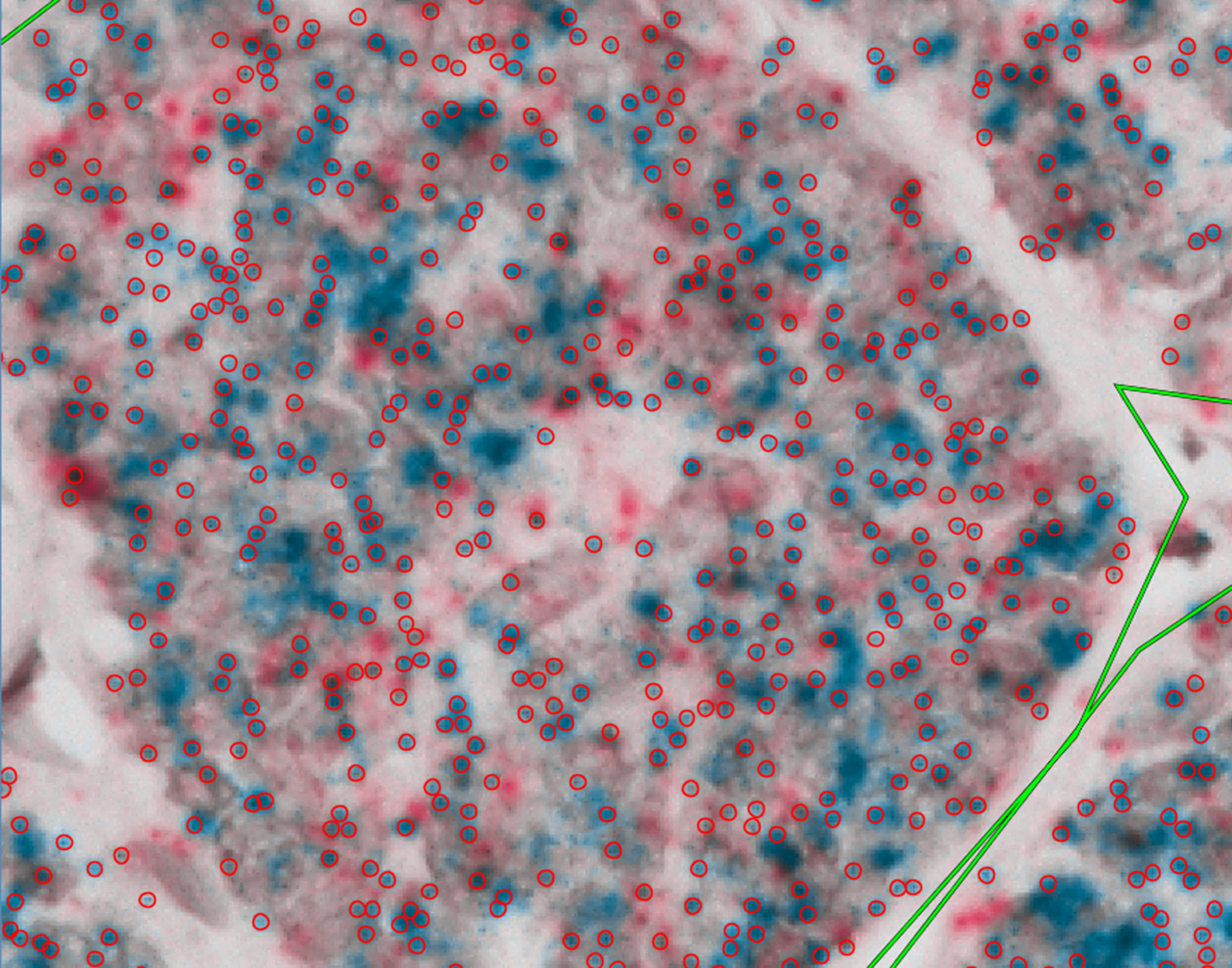

Supplement: S1 Fig — (TIF) [file pone.0229031.s002.tif]
